# Professional, social, and demographic correlates of nursing care rationing: insights from hospital nurses in the Czech Republic, Poland and Slovakia

**DOI:** 10.3389/fmed.2026.1849770

**Published:** 2026-06-19

**Authors:** Krystyna Kowalczuk, Katarzyna Tomaszewska, Helena Kadučáková, Anna Krátká, Erika Durejova, Marek Sobolewski, Justyna Magdalena Hermanowicz

**Affiliations:** 1 Department of Integrated Medical Care, Medical University of Bialystok, Bialystok, Poland; 2 Department of Nursing, Institute of Health Protection, The Bronislaw Markiewicz State Higher School of Technology and Economics, Jaroslaw, Poland; 3 Department of Nursing, Faculty of Health Sciences, Catholic University in Ružomberok, Ružomberok, Slovakia; 4 Department of Health Care Sciences, Faculty of Humanities, Tomas Bata University in Zlin, Zlín, Czechia; 5 Ustredna Vojenska Nemocnica Ružomberok, Ružomberok, Slovakia; 6 Faculty of Management, Rzeszow University of Technology, Rzeszow, Poland; 7 Department of Pharmacodynamics, Medical University of Bialystok, Bialystok, Poland

**Keywords:** care rationing, medical documentation, missed nursing care, nursing staff, quality of healthcare

## Abstract

**Introduction:**

Nursing care rationing is an increasingly common problem in healthcare systems. It results in reduced quality of care, decreased patient satisfaction, and an increased risk of medical errors. The aim of this study was to assess the level and patterns of nursing care rationing and to examine associations between rationing and selected demographic and professional characteristics of hospital nurses in the Czech Republic, Poland and Slovakia.

**Materials and methods:**

A cross-sectional study was conducted between March 2024 and March 2025. The study included 610 licensed nurses working in hospitals in Zlín, Czech Republic (151), Białystok, Poland (245) and Ružomberok, Slovakia (214). The study was conducted using the standardized BERNCA-R scale. The frequency of omitting 32 nursing activities was assessed, and correlations with demographic and professional factors were analyzed. Data were examined using descriptive statistics, Kruskal–Wallis tests, and Spearman’s rank correlations.

**Results:**

The mean overall BERNCA-R score differed slightly between the hospital samples. Demographic and professional characteristics—including age, marital status, parenthood, education, and years of experience—were not significantly associated with the overall level of rationing. In contrast, the type of hospital ward showed a stronger association with rationing patterns, with emergency departments reporting the highest frequency of omitted nursing activities. Analysis of individual BERNCA-R items indicated that activities requiring extended patient communication, assessment, or monitoring were more likely to be rationed, whereas basic hygiene-related tasks were rarely omitted.

**Conclusion:**

The findings suggest that nursing care rationing in hospital settings is more strongly associated with organizational and workload-related factors than with individual nurse characteristics. Efforts to reduce rationing should therefore focus on improving staffing structures, workflow organization, and working conditions, particularly in high-intensity clinical environments.

## 1 Introduction

Contemporary healthcare systems in Central and Eastern Europe, including Poland, the Czech Republic, and Slovakia, face growing challenges related to nursing staff shortages, an aging population, work overload, and limited resources. One of the most concerning consequences of these challenges is the rationing of nursing care (1).

Nursing care rationing refers to situations in which nurses, due to limited resources—such as time, staffing, or organizational constraints—are unable to perform all clinically necessary care activities (2, 3). In the literature, this concept is sometimes used interchangeably with “missed care”, “unfinished nursing care” or “implicit rationing”. It involves the deliberate or forced omission, delay, or reduction of nursing interventions, despite their clinical indication (2). This phenomenon most commonly affects fundamental aspects of care, including patient monitoring, emotional support, health education, and daily nursing activities. Rationing of care typically results from shortages in staffing, time, or organizational support (3).

In 2007, Schubert et al. developed the BERNCA (Basel Extent of Rationing of Nursing Care) questionnaire to measure the intensity of nursing care rationing (2). Studies in Swiss hospitals using BERNCA identified emotional support, patient education, and completion of medical documentation as the care activities most frequently omitted (4).

Key factors influencing nursing care rationing include staffing levels, working conditions, management practices, workload, and occupational stress. Schubert et al. (4) identified a low nurse-to-patient ratio as one of the strongest predictors of care rationing. Hospitals where nurses have limited professional autonomy and operate within hierarchical structures tend to experience higher levels of rationing. In such contexts, nurses are often forced to make difficult decisions, foregoing certain aspects of patient care to meet organizational demands (5).

Individual nurse characteristics—such as age, educational background, professional experience, and type of employment—may also influence their capacity to manage workload and stress, thereby affecting care rationing (6). Younger nurses are often more vulnerable to stress related to work organization, whereas older nurses with longer professional tenure may manage time more effectively but face a higher risk of chronic overload (7). Higher educational attainment is associated with stronger organizational and task-delegation skills, which may enable nurses to manage workloads more efficiently and reduce care rationing (8).

Research by Schubert and Ball et al. (9, 10) demonstrated that nursing care rationing is associated with negative patient outcomes, increased complications, and reduced satisfaction among both patients and nursing staff. It also impacts nurses’ morale and may contribute to professional burnout. Similar studies in Canada (11) confirmed that care rationing is widespread and poses a significant risk to patient safety, increasing the likelihood of medical errors (12), complications such as pressure ulcers, infections, and falls, and negatively affecting nurses’ professional quality of life (13).

Pan-European studies have further investigated this phenomenon. The RN4CAST project, including 12 countries—Poland among them—demonstrated that a low nurse-to-patient ratio strongly predicts care rationing and negative patient outcomes, including increased mortality. Poland ranked among the countries with the highest levels of care rationing (12). The RANCARE project (2016–2019), encompassing 27 European countries, highlighted the importance of considering systemic, cultural, and organizational factors when analyzing nursing care rationing across national contexts (14).

Healthcare systems in Poland, the Czech Republic, and Slovakia differ markedly in organization, financing, and structure. Poland’s system is insurance-based, dominated by the National Health Fund (Narodowy Fundusz Zdrowia), which contracts services with medical facilities and is primarily financed through health insurance contributions paid to the Social Insurance Fund (Zakład Ubezpieczeń Społecznych). The system is centralized with limited provider competition (15). In contrast, the Czech system is decentralized, operating under universal health insurance with competing sickness funds, the largest being the General Health Insurance Fund (Všeobecná Zdravotní Pojišťovna), along with several branch-specific funds. Patients may choose their insurer, introducing competition that can influence service quality (16). Slovakia also relies on insurance-based financing with multiple sickness funds, but competition is less pronounced than in the Czech Republic (17).

Differences in healthcare spending reflect these structural variations. According to recent data, Poland spends approximately 6.4% of GDP on healthcare, Slovakia 7.7%, and the Czech Republic 8.8%, compared to the EU average of 10.2%. Per capita expenditures are €1,137 in Poland, €1,561 in Slovakia, and €2,278 in the Czech Republic, while the EU average is €3,685 (18).

Hospital infrastructure also differs. In 2022, the number of hospital beds per 1,000 inhabitants was highest in the Czech Republic (6.7), followed by Poland (6.1), and lowest in Slovakia (5.7), all above the EU average of 5.2 (19). Despite these figures, hospital types vary: Poland predominantly has small county hospitals facing staffing shortages, while the Czech Republic and Slovakia have larger university hospitals better able to meet patient demand (20).

Workforce indicators further illustrate disparities. In 2023, the Czech Republic reported 792.4 nurses per 100,000 inhabitants, compared to 589.0 in Poland and 574.5 in Slovakia (20). When measured as nurses per hospital bed, Slovakia led with 1 nurse per bed, the Czech Republic had 0.89, and Poland only 0.53—indicating that, on average, one nurse in Poland was responsible for two beds. The EU average was 1.06 nurses per bed (20).

The private sector’s role also varies. In Poland, private hospitals represent a substantial component, operating commercially and non-profit, with many contracted by the National Health Fund. In the Czech Republic and Slovakia, hospitals are predominantly public, managed by state or regional authorities, with private facilities largely limited to specialized or outpatient care (21).

In summary, the Czech healthcare system benefits from stronger financing, a larger workforce, and a more decentralized structure with competitive insurance funds, whereas Poland and Slovakia have more centralized systems with lower funding and greater staffing challenges. Differences in healthcare system organization may contribute to varying levels of nursing care rationing. Studies in Poland highlight task overload, staffing shortages, and occupational stress as primary drivers (22, 23), with age and professional tenure also influencing care rationing. Research from the Czech Republic and Slovakia identifies similar determinants, particularly insufficient staffing and the high pace of clinical work (24).

While prior studies have emphasized structural determinants such as staffing ratios or hospital policies, individual nurse characteristics—including age, education, professional experience, marital status, parenthood, and ward type—may affect how nurses prioritize and deliver care under resource constraints.

This study aimed to examine associations between nursing care rationing and nurses’ demographic, social, and professional characteristics among hospital nurses from three selected hospitals located in Poland, the Czech Republic, and Slovakia. Although the study included samples from three countries, the primary objective was not to compare healthcare systems but to investigate whether individual and workplace-related factors were associated with the reported level of nursing care rationing within each national sample. Specifically, the study sought to:

a) Assess the overall level and patterns of nursing care rationing among hospital nurses.
b) Examine associations between nursing care rationing and nurses’ demographic and professional characteristics, including age, education, years of experience, and hospital unit.
c) Identify specific nursing activities most frequently subject to rationing.

The findings are expected to contribute to international research on nursing care rationing by highlighting the role of workforce-related determinants and providing exploratory evidence relevant for workforce planning and the improvement of nurses’ working conditions in similar hospital settings.

## 2 Materials and methods

A cross-sectional study was conducted between March 2024 and March 2025 in three neighboring countries: the Czech Republic, Poland, and Slovakia. In each country, the study was carried out in a single hospital—specifically in Zlín, Białystok, and Ružomberok. These hospitals were selected because they are comparable in size (number of beds), share the same teaching or university-affiliated status, and offer a similar range of inpatient services. All three institutions are relatively large regional hospitals within their respective healthcare systems and include internal medicine, surgical, and emergency departments. The hospitals were selected to maximize organizational comparability rather than national representativeness.

The research was approved by the appropriate authorities. In Zlín and Ružomberok, approvals were granted by the hospital administrations. In Białystok, approval was obtained from the Bioethics Committee of the Medical University of Białystok (approval no. APK.002.137.2024). All procedures involving human participants complied with international ethical standards, including voluntary participation, informed consent, anonymity, confidentiality, avoidance of harm, and communication of results.

### 2.1 Selection of the study group and study protocol

The inclusion criterion was full-time hospital employment under a contract of employment. Nurses working part-time or under arrangements other than an employment contract were excluded from participation. In each participating hospital, 300 paper questionnaires were distributed to all nurses who met the inclusion criteria and were present at work during the data collection period. Participation was voluntary, anonymous, and could be withdrawn at any time without providing a reason. Nurses were asked to complete the surveys in their free time and return them in sealed envelopes.

Of the 900 questionnaires distributed, 610 were returned correctly completed, yielding an overall response rate of 67.8%. The response rates by country were as follows: Poland 81.6%, Slovakia 71.3%, and the Czech Republic 50.0%. The final sample comprised 610 licensed nurses from hospitals in Zlín, Czech Republic (*n* = 151), Białystok, Poland (*n* = 245), and Ružomberok, Slovakia (*n* = 214). Differences in sample size across the three countries resulted solely from variations in the number of questionnaires returned, as participation was voluntary and the reasons for nonresponse are unknown. All demographic and professional data were obtained through self-report, and no incentives were offered.

### 2.2 Description of questionnaires and measures

To assess nursing care rationing, we used the standardized BERNCA-R questionnaire (25, 26). A validated Polish version of the instrument served as the basis for the study. The Czech and Slovak versions were translated from the validated Polish version by bilingual researchers experienced in nursing and healthcare research. The translated versions were subsequently reviewed jointly by the international research team to ensure conceptual consistency and cultural appropriateness of the questionnaire items within local clinical contexts. The adaptation process focused on preserving the meaning and clinical relevance of individual nursing activities across all language versions. However, a formal forward–backward translation procedure and separate pilot psychometric validation were not performed prior to the study, which should be considered when interpreting the findings. Permission to use the BERNCA-R scale was obtained from the original developers prior to data collection.

The questionnaire consists of 32 detailed statements rated on a five-point adjectival scale. Responses are coded from 0 to 4, so the total rationing measure can range from 0 to 128 points, with higher values being unfavorable and indicating greater limitations in nursing care provided to patients.

### 2.3 Statistical methods

First, analyses were conducted separately for the Polish, Slovak, and Czech samples. The analysis involved compiling selected descriptive statistics (mean values with 95% confidence intervals) of the overall BERNCA-R scores in relation to levels of grouping factors and assessing the significance of differences. Differences between groups were tested using Kruskal-Wallis test. A nonparametric test was used because the distribution of BERNCA-R scores showed slight skewness and the results of the Shapiro–Wilk test in the compared groups were statistically significant, indicating a lack of normality in the distribution. For ordinal variables, such as age and years of experience, an alternative approach was also applied by calculating Spearman rank correlations between these factors and the psychometric measures.

For the comparative analysis of responses to the individual items of the BERNCA-R questionnaire, the Kruskal-Wallis test was used to assess the significance of differences in the level of care rationing reported by nurses from the different countries. To facilitate interpretation, statistical significance was marked as follows: *p* < 0.05 (*), *p* < 0.01 (**), *p* < 0.001 (***).

## 3 Results

### 3.1 Characteristics of the study groups

Women clearly predominated among respondents, accounting for approximately 90% of the nursing staff in all three samples (*p* < 0.001). Due to this strong gender imbalance, sex was not included in further analyses.

The age distribution of nurses was similar across the three samples (*p* > 0.001). In each group, married nurses constituted the largest proportion of respondents, although the Czech sample showed the lowest proportion of married participants and the highest proportion of nurses living in informal partnerships (28.7%).

Approximately half of the respondents reported having children. However, in the Slovak sample more than one-third of participants did not provide information on this variable.

Differences were observed in educational attainment and ward distribution. The Polish sample included the highest proportion of nurses with master’s degrees (*p* < 0.001). In addition, a relatively smaller proportion of Polish nurses worked in internal medicine wards and a larger proportion worked in surgical wards compared with the other two samples (*p* < 0.001).

The predominance of female nurses and the age distribution observed in the study sample are generally consistent with national nursing workforce characteristics reported in Central European countries, where nursing remains a predominantly female profession with an aging workforce structure. A comparative profile of the three study samples is presented in Table 1.

**Table 1:** Comparative characteristics of the samples.

Characteristics/values		Country					
		Slovakia		Poland		Czech Rep.	
		*N*	%_↓_	*N*	%_↓_	*N*	%_↓_
Gender	Female	205	95.8%	219	89.4%	143	96.6%
	Male	9	4.2%	26	10.6%	5	3.4%
Age [years]	<28	33	15.4%	54	22.0%	36	24.5%
	28–33	16	7.5%	38	15.5%	13	8.8%
	34–39	24	11.2%	27	11.0%	13	8.8%
	40–45	52	24.3%	35	14.3%	29	19.7%
	46–51	37	17.3%	49	20.0%	31	21.1%
	52–57	33	15.4%	33	13.5%	22	15.0%
	>57	19	8.9%	9	3.7%	3	2.0%
Marital status	Partnership	46	21.5%	59	24.1%	41	28.7%
	Married	137	64.0%	163	66.5%	76	53.1%
	Single	31	14.5%	23	9.4%	26	18.2%
Children	Yes	104	48.6%	132	53.9%	84	55.6%
	No	36	16.8%	94	38.4%	51	33.8%
	No response	74	34.6%	19	7.8%	16	10.6%
Education	Nursing diploma	40	18.7%	34	13.9%	80	54.4%
	Bachelor’s degree	92	43.0%	85	34.7%	57	38.8%
	Master’s degree	82	38.3%	126	51.4%	10	6.8%
Years of experience	<6	57	26.6%	82	33.5%	45	31.5%
	6–11	43	20.1%	46	18.8%	24	16.8%
	12–17	30	14.0%	30	12.2%	21	14.7%
	18–23	35	16.4%	53	21.6%	31	21.7%
	>23	49	22.9%	34	13.9%	22	15.4%
Hospital ward	Internal medicine	115	59.3%	90	36.7%	78	59.5%
	Surgical	66	34.0%	102	41.6%	25	19.1%
	Emergency	13	6.7%	53	21.6%	28	21.4%

### 3.2 Aggregate nursing care rationing measures

Table 2 presents the distribution of overall BERNCA-R scores in the Polish, Slovak, and Czech samples. Mean values with 95% confidence intervals are shown.

**Table 2:** Distribution of the overall BERNCA-R nursing care rationing measure.

Country	Nursing care rationing (*p* = 0.0000***)					
	*N*	Mean (95% CI)	Median	Std. dev.	Min	Max
Slovakia	214	41.1 (38.4–43.8)	39	19.8	0	95
Poland	245	48.4 (46.3–50.6)	48	16.9	9	93
Czech Rep.	151	49.0 (46.2–51.7)	47	17.1	0	111

*p*, probability value calculated using Kruskal-Wallis test. *p<0.05, **p<0.01, ***p<0.001.

The Kruskal–Wallis test indicated statistically significant differences in overall rationing scores between the national samples (*p* < 0.001).

### 3.3 Demographic and professional factors and overall BERNCA-R scores

The analysis examined whether selected demographic (age, marital status, parenthood) and professional (education, work experience, hospital unit) characteristics were associated with the level of nursing care rationing. No statistically significant differences were observed across age groups, marital status, parenthood status, educational level, or years of professional experience in any of the three samples (*p* > 0.05 for all comparisons, Kruskal–Wallis test).

A statistically significant association was observed only in the Slovak sample, where nurses working in emergency departments reported higher levels of care rationing compared with those working in other hospital units (*p* < 0.001). This pattern was not observed in the Polish or Czech samples (*p* > 0.05) (Table 3).

**Table 3:** Nursing care rationing by hospital ward type.

Ward	Nursing care rationing					
	Slovakia		Poland		Czech Rep.	
	*N*	Mean (95% CI)	*N*	Mean (95% CI)	*N*	Mean (95% CI)
Internal medical	115	41.3 (37.6–45.1)	90	46.3 (42.7–49.8)	78	51.3 (47.6–54.9)
Surgical	66	36.3 (32.3–40.2)	102	50.5 (47.1–53.8)	25	48.1 (41.0–55.2)
Emergency	13	62.5 (50.8–74.2)	53	48.3 (43.7–52.9)	28	50.0 (42.3–57.8)
*p*	0.0003***		0.1443		0.7209	

*p*, probability value calculated using Kruskal-Wallis test.

Because differences in the reported need to ration nursing care were observed across hospital units, an additional analysis was conducted to examine whether the clinical context of work (type of hospital unit) could influence the observed rationing levels. Mean overall BERNCA-R scores with 95% confidence intervals were therefore examined within each unit type. Detailed results are presented in Table 4.

**Table 4:** Nursing care rationing in specific wards by country groups.

Country	Nursing care rationing					
	Hospital ward					
	Internal medical		Surgical		Emergency	
	*N*	Mean (95% CI)	*N*	Mean (95% CI)	*N*	Mean (95% CI)
Slovakia	115	41.3 (37.6–45.1)	66	36.3 (32.3–40.2)	13	62.5 (50.8–74.2)
Poland	90	46.3 (42.7–49.8)	102	50.5 (47.1–53.8)	53	48.3 (43.7–52.9)
Czech Rep.	78	51.3 (47.6–54.9)	25	48.1 (41.0–55.2)	28	50.0 (42.3–57.8)
*p*	0.0013**		0.0000***		0.0928	

*p*, probability value calculated using Kruskal-Wallis test. *p<0.05, **p<0.01, ***p<0.001.

### 3.4 Specific aspects of nursing care rationing

A comparative analysis of responses to individual items of the BERNCA-R questionnaire was conducted to identify which nursing activities were most frequently subject to rationing. Mean rationing scores with 95% confidence intervals were calculated for each questionnaire item within each national sample. Differences between samples were assessed using the Kruskal–Wallis test. The results are presented in Table 5.

**Table 5:** Mean assessment of the necessity for nursing care rationing.

Nursing care items	Mean assessment of necessity for rationing			*p*
	Slovakia	Poland	Czech Rep.	
Assisting with a complete bed bath	1.04	1.20	1.50	0.0001***
Providing necessary daily washing or toileting in bed	1.05	1.25	1.36	0.0072**
Performing skin care to prevent irritation or pressure injuries	1.11	1.36	1.42	0.0006***
Assisting with oral hygiene (mouth care)	1.10	1.38	1.47	0.0000***
Supporting with tooth brushing or dental hygiene	1.04	1.46	1.47	0.0000***
Helping during meals or feeding	1.17	1.26	1.41	0.0068**
Assisting with mobility and movement	1.35	1.44	1.52	0.1369
Helping change position in bed	1.38	1.45	1.51	0.3168
Changing soiled linen and clothing promptly	1.29	1.55	1.62	0.0010***
Offering emotional support	1.40	1.61	1.65	0.0239*
Talking with patients or family members	1.57	1.78	1.73	0.0921
Giving explanations and information about their care	1.38	1.66	1.62	0.0020**
Assisting to use the toilet or providing incontinence care	1.27	1.48	1.61	0.0036**
Assisting with catheter care	1.34	1.33	1.55	0.0234*
Engaging patients in activities that stimulate or activate them	1.42	1.46	1.69	0.0110*
Educating patients about managing diabetes	1.24	1.47	1.45	0.0293*
Preparing patients for hospital discharge	1.18	1.51	1.53	0.0001***
Carrying out checks according to physicians’ orders	1.38	1.65	1.68	0.0003***
Performing checks on own assessment of patient condition	1.47	1.78	1.74	0.0004***
Monitoring immobilized patients	1.38	1.46	1.77	0.0015**
Monitoring patients under sedatives or calming medication	1.39	1.51	1.75	0.0051**
Delaying care because of waiting for a physician	1.24	1.53	1.44	0.0110*
Administering medication at the correct time	1.29	1.59	1.78	0.0000***
Providing wound care without delay	1.08	1.52	1.38	0.0000***
Preparing patients for diagnostic tests	1.15	1.47	1.36	0.0000***
Responding quickly when patients call	1.22	1.53	1.36	0.0013**
Practicing proper hand hygiene	1.17	1.37	1.28	0.0315*
Performing disinfection	1.15	1.30	1.26	0.1346
Familiarizing with patient condition	1.42	1.85	1.59	0.0000***
Assessing the needs of newly admitted patients	1.48	1.82	1.46	0.0000***
Developing an individualized nursing care plan	1.38	1.78	1.50	0.0000***
Completing nursing documentation	1.38	1.65	1.50	0.0152*

*p*, probability value calculated using Kruskal-Wallis test. *p<0.05, **p<0.01, ***p<0.001.

### 3.5 Correlations of demographic and professional factors with individual items of BERNCA-R

#### 3.5.1 Age and nursing care rationing items

Only a small number of statistically significant correlations between age and individual rationing items were observed, and all were weak (absolute correlation coefficient below 0.30). Most of these correlations were found in the Czech sample and involved tasks such as promptly changing soiled linen and clothing, delaying care due to waiting for a physician, administering medications at the correct time, and preparing patients for diagnostic tests. The fewest correlations were observed in the Slovak sample, where only one task—helping patients change position in bed—showed a significant association.

All identified correlations were negative, indicating that older nurses were less likely to report rationing specific care activities. Detailed results are presented in Table 6.

**Table 6:** Spearman’s rank correlation between the assessment of the necessity for rationing nursing care items and nurses’ age.

Nursing care items	Slovakia	Poland	Czech Rep.
	Age [years]		
Assisting with a complete bed bath	0.05	0.04	0.04
Providing necessary daily washing or toileting in bed	−0.01	0.01	−0.05
Performing skin care to prevent irritation or pressure injuries	−0.01	−0.01	−0.03
Assisting with oral hygiene (mouth care)	−0.06	−0.05	0.11
Supporting with tooth brushing or dental hygiene	−0.04	−0.02	0.05
Helping during meals or feeding	−0.10	−0.02	0.14
Assisting with mobility and movement	−0.04	−0.02	0.00
Helping change position in bed	−0.15*	0.04	0.03
Changing soiled linen and clothing promptly	−0.04	−0.01	−0.19*
Offering emotional support	0.01	0.03	−0.04
Talking with patients or family members	0.05	0.00	−0.06
Giving explanations and information about their care	−0.03	0.01	−0.08
Assisting to use the toilet or providing incontinence care	−0.03	−0.01	0.01
Assisting with catheter care	0.03	0.06	0.00
Engaging patients in activities that stimulate or activate them	−0.05	0.10	0.02
Educating patients about managing diabetes	0.03	0.07	0.05
Preparing patients for hospital discharge	−0.05	0.02	0.01
Carrying out checks according to physicians’ orders	0.02	−0.13*	−0.14
Performing checks on own assessment of patient condition	0.02	−0.12	−0.10
Monitoring immobilized patients	0.05	0.02	−0.09
Monitoring patients under sedatives or calming medication	0.04	0.09	0.01
Delaying care because of waiting for a physician	0.00	0.03	−0.19*
Administering medication at the correct time	−0.09	−0.21*	−0.25*
Providing wound care without delay	0.02	−0.15*	−0.02
Preparing patients for diagnostic tests	0.03	0.00	−0.19*
Responding quickly when patients call	−0.07	−0.03	−0.16
Practicing proper hand hygiene	−0.03	−0.06	0.01
Performing disinfection	0.00	−0.12	−0.01
Familiarizing with patient condition	0.02	−0.12	−0.11
Assessing the needs of newly admitted patients	0.03	−0.11	−0.05
Developing an individualized nursing care plan	−0.02	−0.04	−0.14
Completing nursing documentation	0.01	−0.02	−0.15

* Significance correlations (*p* < 0.05).

#### 3.5.2 Work experience and nursing care rationing items

Very weak correlations between years of professional experience and rationing of specific care activities were observed only in the Polish sample. Three statistically significant correlations were identified: administering medication at the correct time (*p* = −0.16), assessing the needs of newly admitted patients (*p* = −0.15), and providing wound care without delay (*p* = −0.15).

#### 3.5.3 Education and nursing care rationing items

No significant differences in rationing assessments were found by education level among Slovak nurses. In the Polish sample, two exceptions were identified: nurses with higher education rationed emotional support less frequently (means: 1.74, 1.80, and 1.45 – *p* = 0.0414*), but more often delayed care while waiting for a physician (means: 1.35, 1.33, and 1.71 – *p* = 0.02518*). In the Czech sample, one exception was observed: nurses with higher education rated the rationing of patient assessments significantly differently (means: 1.76, 1.84, and 1.00 – *p* = 0.0173*).

#### 3.5.4 Type of hospital ward and nursing care rationing items

The type of hospital ward was associated with differences in rationing assessments for a relatively large number of questionnaire items in the Slovak sample (0.001 < *p* < 0.1). Overall, nurses working in emergency departments reported the highest number of rationed care activities, whereas the lowest levels were reported by nurses working in surgical wards. Detailed results are presented in Table 7.

**Table 7:** The relationship between the type of hospital ward and items of nursing care rationing in the Slovak sample.

Nursing care items	Mean assessment of necessity for rationing depending on hospital ward			*p*
	Internal medicine	Surgical	Emergency	
Assisting with a complete bed bath	1.14	0.67	1.36	0.0002***
Providing necessary daily washing or toileting in bed	1.11	0.79	1.21	0.0095**
Performing skin care to prevent irritation or pressure injuries	1.23	0.77	1.43	0.0005***
Assisting with oral hygiene (mouth care)	1.17	0.85	1.36	0.0244*
Supporting with tooth brushing or dental hygiene	1.12	0.77	1.32	0.0192*
Helping during meals or feeding	1.29	0.91	1.39	0.0403*
Assisting with mobility and movement	1.28	1.35	1.68	0.3740
Helping change position in bed	1.29	1.45	1.46	0.6028
Changing soiled linen and clothing promptly	1.25	1.29	1.50	0.9006
Offering emotional support	1.43	1.39	1.39	0.8373
Talking with patients or family members	1.62	1.33	1.86	0.0874
Giving explanations and information about their care	1.44	1.30	1.32	0.6175
Assisting to use the toilet or providing incontinence care	1.35	1.12	1.50	0.3720
Assisting with catheter care	1.41	1.06	1.68	0.0430*
Engaging patients in activities that stimulate or activate them	1.44	1.26	1.71	0.3438
Educating patients about managing diabetes	1.31	0.91	1.64	0.0071**
Preparing patients for hospital discharge	1.31	1.02	0.89	0.0089**
Carrying out checks according to physicians’ orders	1.46	1.24	1.46	0.2259
Performing checks on own assessment of patient condition	1.54	1.32	1.64	0.2431
Monitoring immobilized patients	1.42	1.11	1.79	0.0638
Monitoring patients under sedatives or calming medication	1.39	1.15	1.82	0.0910
Delaying care because of waiting for a physician	1.35	1.15	1.14	0.1088
Administering medication at the correct time	1.34	1.30	1.21	0.3540
Providing wound care without delay	1.11	0.98	1.07	0.7622
Preparing patients for diagnostic tests	1.32	0.97	1.07	0.0155*
Responding quickly when patients call	1.28	1.27	0.96	0.1166
Practicing proper hand hygiene	1.15	1.15	1.29	0.5206
Performing disinfection	1.17	1.08	1.00	0.1519
Familiarizing with patient condition	1.55	1.24	1.36	0.0213*
Assessing the needs of newly admitted patients	1.57	1.42	1.43	0.5013
Developing an individualized nursing care plan	1.53	1.29	1.25	0.0830
Completing nursing documentation	1.48	1.35	1.04	0.0130*

*p*, probability value calculated using Kruskal-Wallis test.

Among Polish nurses, fewer differences between wards were observed compared with the Slovak sample. For the limited number of items showing statistically significant variation, higher levels of rationing were again reported by nurses working in emergency departments (0.001 < *p* < 0.1). Detailed results are presented in Table 8.

**Table 8:** The relationship between the type of hospital ward and items of nursing care rationing in the Polish sample.

Nursing care items	Mean assessment of necessity for rationing depending on hospital ward			*p*
	Internal medicine	Surgical	Emergency	
Assisting with a complete bed bath	1.12	1.38	1.00	0.0606
Providing necessary daily washing or toileting in bed	1.14	1.42	1.11	0.1255
Performing skin care to prevent irritation or pressure injuries	1.29	1.49	1.21	0.0849
Assisting with oral hygiene (mouth care)	1.34	1.52	1.19	0.1072
Supporting with tooth brushing or dental hygiene	1.42	1.61	1.23	0.0693
Helping during meals or feeding	1.14	1.35	1.28	0.3311
Assisting with mobility and movement	1.54	1.45	1.25	0.2212
Helping change position in bed	1.42	1.59	1.23	0.1012
Changing soiled linen and clothing promptly	1.48	1.62	1.55	0.6606
Offering emotional support	1.64	1.64	1.51	0.8448
Talking with patients or family members	1.66	1.82	1.89	0.3585
Giving explanations and information about their care	1.57	1.65	1.83	0.2788
Assisting to use the toilet or providing incontinence care	1.41	1.57	1.43	0.5627
Assisting with catheter care	1.27	1.51	1.08	0.0805
Engaging patients in activities that stimulate or activate them	1.33	1.54	1.51	0.4251
Educating patients about managing diabetes	1.29	1.57	1.57	0.1086
Preparing patients for hospital discharge	1.54	1.47	1.53	0.9834
Carrying out checks according to physicians’ orders	1.57	1.75	1.60	0.2353
Performing checks on own assessment of patient condition	1.69	1.81	1.89	0.2451
Monitoring immobilized patients	1.29	1.58	1.53	0.2289
Monitoring patients under sedatives or calming medication	1.41	1.64	1.42	0.3557
Delaying care because of waiting for a physician	1.57	1.55	1.43	0.8537
Administering medication at the correct time	1.61	1.51	1.72	0.3844
Providing wound care without delay	1.42	1.48	1.75	0.0688
Preparing patients for diagnostic tests	1.36	1.60	1.40	0.0462*
Responding quickly when patients call	1.64	1.54	1.30	0.1592
Practicing proper hand hygiene	1.37	1.32	1.45	0.7797
Performing disinfection	1.29	1.17	1.57	0.0113*
Familiarizing with patient condition	1.66	1.90	2.09	0.0094**
Assessing the needs of newly admitted patients	1.60	1.91	2.04	0.0127*
Developing an individualized nursing care plan	1.61	1.77	2.06	0.0167*
Completing nursing documentation	1.56	1.73	1.66	0.6469

*p*, probability value calculated using Kruskal-Wallis test. *p<0.05, **p<0.01, ***p<0.001.

In the Czech sample, statistically significant differences between wards were observed for only two items (0.001 < *p* < 0.1). These items referred to oral and dental hygiene, with nurses in surgical wards reporting lower levels of rationing compared with other units. Detailed results are presented in Table 9.

**Table 9:** The relationship between the type of hospital ward and items of nursing care rationing in the Czech sample.

Nursing care items	Mean assessment of necessity for rationing depending on hospital ward			*p*
	Internal medicine	Surgical	Emergency	
Assisting with a complete bed bath	1.60	1.44	1.43	0.5500
Providing necessary daily washing or toileting in bed	1.51	1.24	1.32	0.2556
Performing skin care to prevent irritation or pressure injuries	1.59	1.24	1.46	0.2339
Assisting with oral hygiene (mouth care)	1.65	1.00	1.54	0.0104*
Supporting with tooth brushing or dental hygiene	1.72	1.16	1.36	0.0064**
Helping during meals or feeding	1.56	1.20	1.39	0.1581
Assisting with mobility and movement	1.64	1.64	1.43	0.5426
Helping change position in bed	1.60	1.40	1.57	0.4617
Changing soiled linen and clothing promptly	1.68	1.72	1.61	0.8525
Offering emotional support	1.72	1.60	1.68	0.9547
Talking with patients or family members	1.74	1.72	1.89	0.6250
Giving explanations and information about their care	1.68	1.64	1.57	0.9748
Assisting to use the toilet or providing incontinence care	1.65	1.56	1.79	0.6671
Assisting with catheter care	1.62	1.48	1.64	0.8841
Engaging patients in activities that stimulate or activate them	1.69	1.56	1.89	0.5418
Educating patients about managing diabetes	1.37	1.72	1.50	0.2218
Preparing patients for hospital discharge	1.60	1.40	1.57	0.6664
Carrying out checks according to physicians’ orders	1.76	1.68	1.79	0.8855
Performing checks on own assessment of patient condition	1.83	1.68	1.82	0.7864
Monitoring immobilized patients	1.76	1.68	2.07	0.4507
Monitoring patients under sedatives or calming medication	1.79	1.48	2.18	0.0883
Delaying care because of waiting for a physician	1.51	1.60	1.36	0.4776
Administering medication at the correct time	1.94	1.64	1.79	0.5661
Providing wound care without delay	1.40	1.48	1.43	0.8683
Preparing patients for diagnostic tests	1.37	1.52	1.36	0.5424
Responding quickly when patients call	1.46	1.44	1.39	0.9179
Practicing proper hand hygiene	1.29	1.44	1.21	0.4017
Performing disinfection	1.26	1.40	1.32	0.6233
Familiarizing with patient condition	1.56	1.72	1.68	0.7267
Assessing the needs of newly admitted patients	1.55	1.36	1.39	0.4572
Developing an individualized nursing care plan	1.56	1.76	1.29	0.1679
Completing nursing documentation	1.59	1.48	1.32	0.2712

*p*, probability value calculated using Kruskal-Wallis test.

## 4 Discussion

The aim of this study was to examine the level and patterns of nursing care rationing in hospital settings and to explore whether selected demographic and professional characteristics of nurses were associated with the reported level of rationing. The study included nurses from three hospitals located in Central European countries with broadly similar historical and socio-economic contexts; however, the primary objective was not to compare national healthcare systems but to explore patterns of care rationing within a broader regional nursing workforce.

The results indicate that nursing care rationing occurred in all study samples, although the reported levels differed between them. The mean value of the overall BERNCA-R index was lowest in the Slovak sample (41.1 points), higher in the Polish sample (48.4 points), and highest in the Czech sample (49.0 points). Although these differences were statistically significant, they should be interpreted with caution. Because only one hospital per country participated in the study, system-level characteristics were not included as analytic variables and could not be statistically modeled. Consequently, the observed variation between samples may reflect institutional or contextual factors specific to the participating hospitals rather than differences between national healthcare systems. Because only one hospital from each country participated in the study, the findings should not be interpreted as representative of national nursing populations or healthcare systems. The observed differences are more appropriately understood as reflecting characteristics of the participating institutions and their organizational environments.

The relatively higher level of reported rationing in the Czech sample may therefore be related to local organizational conditions rather than broader structural features of the Czech healthcare system. Nevertheless, healthcare-system characteristics may also indirectly influence nurses’ working conditions. Although the Czech Republic reports higher healthcare expenditures per capita and higher nurse staffing levels compared with Poland and Slovakia, Czech hospitals also operate within highly specialized and intensive inpatient care structures, which may increase workload complexity and documentation burden. Such organizational characteristics could potentially contribute to the higher reported levels of rationing observed in the present study. Previous studies indicate that nursing care rationing is strongly influenced by organizational factors such as workload, staffing allocation, workflow organization, and management practices (4, 12). These factors were not directly measured in the present study but may have influenced the observed results.

Conversely, the lowest levels of rationing were observed in the Slovak sample. One possible explanation may be related to workforce distribution indicators. Although Slovakia reports relatively low numbers of nurses per population, the number of nurses per hospital bed is comparatively higher than in Poland and similar to EU averages. This may potentially reduce workload intensity at the ward level and facilitate the completion of routine nursing activities. However, because institutional staffing indicators were not directly measured, this interpretation remains speculative. However, other contextual factors—such as hospital size, patient acuity, leadership practices, or organizational culture—may also contribute to these findings and were not directly examined in this study.

In the Polish sample, the reported level of rationing was intermediate between the Slovak and Czech samples. This finding supports previous research indicating that nursing care rationing is a multifactorial phenomenon resulting from complex interactions among organizational conditions, workload, staffing structures, and contextual characteristics of hospital environments (12, 14, 27).

The second stage of the analysis examined whether demographic and professional characteristics of nurses were associated with the overall level of nursing care rationing. Across all three samples, no statistically significant differences were observed with respect to age, marital status, parenthood, educational level, or years of professional experience. This finding differs somewhat from earlier studies suggesting that younger age or shorter professional experience may be associated with higher levels of care rationing (28), while higher educational attainment has been linked to lower levels of rationing (29, 30).

One possible explanation for the absence of such associations in the present study may be the relatively homogeneous profile of the respondents. All participants were employed as full-time nurses in large university hospitals, which may have limited the variability of demographic and professional characteristics and thus reduced the ability to detect potential associations.

The type of hospital ward appeared to play a more important role than individual nurse characteristics. Across all samples, the highest levels of rationing were reported in emergency departments, although statistically significant results were observed only in the Slovak sample. Emergency departments are characterized by a rapid pace of work, high patient turnover, and unpredictability of tasks, which can increase the likelihood that some nursing activities are delayed or omitted (10, 31).

To further explore the influence of the clinical work environment, additional analyses examined rationing patterns within specific hospital wards. These analyses confirmed that the reported level of rationing varied across ward types, suggesting that organizational and workload-related factors may influence the extent to which nursing activities are completed as planned.

Although the overall BERNCA-R index provides a useful summary measure, analysis of individual questionnaire items offered additional insight into which nursing activities were most frequently omitted. In the Slovak sample, the most commonly rationed activities involved communication and interaction with patients and families, as well as the assessment of newly admitted patients. In contrast, basic hygiene-related tasks were rarely omitted. This pattern suggests that when time and staffing resources are limited, nurses tend to prioritize essential physiological care over activities requiring extended interpersonal interaction. Similar findings have been reported in studies conducted in other European countries (24, 32).

In the Polish sample, the most frequently rationed activities were related to patient assessment and care planning, including familiarizing oneself with the patient’s condition, assessing the needs of newly admitted patients, and developing individualized care plans. These tasks require time, concentration, and analytical effort, which may make them particularly vulnerable to omission under conditions of high workload. Similar patterns were identified in the RN4CAST project and in studies using the MISSCARE Survey in Poland (33, 34).

In the Czech sample, the most frequently omitted activities were related to timely medication administration and patient monitoring, particularly among immobilized patients or those receiving sedative medication. These findings align with previous research conducted in Czech healthcare settings, which also reported high levels of omission in monitoring-related tasks (24, 35). In contrast, infection-prevention procedures such as hand hygiene and disinfection were rarely omitted, suggesting a strong safety culture in this area.

The final stage of the analysis examined associations between demographic-professional characteristics and the rationing of individual nursing activities. Overall, only a small number of statistically significant correlations were observed, and all were weak. Age showed a small negative association with a few tasks, indicating that older nurses tended to report slightly lower levels of omission for certain activities. However, age was not a strong predictor of rationing overall. These findings differ somewhat from those reported by Mainz et al., who observed higher levels of missed care among younger nurses (7).

Similarly, years of professional experience showed almost no association with rationing behavior, with only a few weak correlations observed in the Polish sample. Previous research conducted by Schubert et al. also reported relatively modest associations between experience and rationing behavior, suggesting that organizational factors may play a more substantial role than individual nurse characteristics (4).

Educational level was also only weakly associated with rationing patterns. While a few isolated differences were observed in the Polish and Czech samples, the overall findings suggest that education alone does not strongly determine the likelihood of rationing specific nursing activities.

The type of hospital ward showed the strongest association with the rationing of individual nursing tasks, particularly in the Slovak sample. Emergency departments consistently demonstrated the highest number of rationed activities, while surgical wards showed the lowest levels. Similar observations have been reported by Kalisch, who emphasized that high-intensity clinical environments tend to prioritize urgent medical tasks at the expense of supportive and non-urgent nursing activities (5).

Overall, the findings suggest that nursing care rationing is more strongly associated with organizational and workload-related conditions than with individual demographic characteristics of nurses. Nevertheless, several potentially relevant variables—such as patient acuity, staffing ratios during individual shifts, managerial support, or organizational culture—were not measured and may have influenced the observed patterns. Because of the cross-sectional design and the limited number of participating institutions, causal conclusions cannot be drawn. Future research should incorporate longitudinal designs and include a broader range of organizational variables in order to better understand the mechanisms underlying nursing care rationing.

## 5 Conclusion

1. Nursing care rationing was reported in all examined hospital settings, indicating that the omission or delay of selected nursing activities occurs in everyday clinical practice. Although the overall level of rationing varied between the study samples, these differences should be interpreted cautiously because the study included only one hospital from each country.
2. Demographic and professional characteristics of nurses—such as age, marital status, parenthood, education, and years of professional experience—were not significantly associated with the overall level of nursing care rationing. These findings suggest that individual nurse characteristics play a limited role in explaining the occurrence of rationing.
3. In the participating hospital samples, organizational context, particularly the type of hospital ward, showed a stronger association with rationing patterns. Emergency departments tended to report higher levels of omitted or delayed nursing activities, indicating that high-intensity clinical environments may increase the likelihood of care rationing.
4. Analysis of individual BERNCA-R items showed that nursing activities requiring time-intensive communication, patient assessment, or monitoring were more likely to be rationed, whereas basic hygiene-related procedures were generally maintained even under conditions of limited time or workload.
5. These findings suggest that, in similar hospital settings, strategies aimed at reducing nursing care rationing should primarily address organizational and workload-related factors, including staffing structures and work organization in high-demand hospital units.
6. From a practical perspective, interventions aimed at reducing nursing care rationing should focus particularly on high-intensity hospital units, including emergency departments. Potential organizational strategies may include optimizing nurse staffing allocation, improving workflow organization, reducing administrative burden, and strengthening managerial support for nursing staff. Some interventions, such as workflow redesign or redistribution of non-clinical tasks, may be implemented with relatively limited financial resources, whereas increasing staffing levels may require substantial long-term investment and healthcare-policy support.

## 6 Methodological limitations

This study has several important limitations that should be taken into account when interpreting the results. The cross-sectional design limits the ability to infer causal relationships between the examined variables. The analysis allows only the identification of statistical associations rather than causal mechanisms. Consequently, it cannot be determined whether the observed demographic or professional characteristics influenced nursing care rationing or whether other unmeasured organizational factors contributed to the reported patterns. The observed associations should therefore be interpreted cautiously and not as evidence of direct causal effects.

The study relied on self-reported data obtained through questionnaires. Such data may be affected by recall bias or social desirability bias, as respondents may underreport or overreport certain behaviors based on perceived professional expectations. Although several demographic and professional variables were included in the analysis, many potentially important organizational factors—such as patient acuity, nurse-to-patient ratios during individual shifts, workload intensity, management practices, leadership support, workflow organization, and workplace culture—were not measured and may have acted as confounding variables. These factors are known to substantially influence nursing workload and prioritization of care activities and therefore may have affected the observed patterns of care rationing in the participating hospitals.

Finally, no formal *a priori* power analysis was conducted because the study was exploratory in nature. An additional limitation is that only one hospital from each country was included in the study. Consequently, the results cannot be considered nationally representative for Poland, the Czech Republic, or Slovakia. Institutional and organizational characteristics specific to the participating hospitals may have influenced the observed patterns of nursing care rationing. Although the sample size was sufficient to detect moderate differences, smaller effects—particularly in subgroup analyses—may not have been detected.

Future research should employ longitudinal and multicenter designs in order to better capture temporal changes and organizational determinants of nursing care rationing. Incorporating institutional-level variables, patient-acuity measures, staffing indicators, and measures of organizational climate may provide a more comprehensive understanding of the mechanisms underlying missed nursing care.

## Data Availability

The raw data supporting the conclusions of this article will be made available by the authors, without undue reservation.
